# Prognostic value of the neutrophil-to-lymphocyte ratio in patients with colorectal cancer undergoing chemotherapy: a systematic review and meta-analysis

**DOI:** 10.3389/fonc.2026.1945549

**Published:** 2026-09-28

**Authors:** Yihui Zhang, Ning Ding, Yingchao Cui, Changlin Yi, Wenkai Chen

**Affiliations:** Department of Laboratory Medicine, Ruijin Hospital, Shanghai Jiao Tong University School of Medicine, Shanghai, China

**Keywords:** chemotherapy, colorectal cancer, neutrophil-to-lymphocyte ratio, prognostic biomarker, survival outcome, systematic review

## Abstract

**Background:**

Colorectal cancer (CRC) remains a major global health burden. Chemotherapy is central to the management of advanced or metastatic disease and is also used in perioperative settings, but outcomes vary substantially. The neutrophil-to-lymphocyte ratio (NLR) is an inexpensive and readily available marker of systemic inflammation; its prognostic association in chemotherapy-treated CRC warrants focused evaluation.

**Methods:**

This study adhered to PRISMA guidelines and was registered with PROSPERO. PubMed, Embase, Web of Science, and the Cochrane Library were searched from inception to July 15, 2025. Studies evaluating pretreatment NLR in relation to overall survival (OS) or a secondary progression- or recurrence-related time-to-event outcome, including progression-free survival (PFS), disease-free survival (DFS), or recurrence-free survival (RFS), in chemotherapy-treated CRC patients were eligible. Study screening, data extraction, and quality assessment were independently conducted by two reviewers. Pooled hazard ratios (HRs) and 95% confidence intervals (CIs) were calculated using random-effects models. Subgroup and leave-one-out sensitivity analyses were performed.

**Results:**

Thirty-eight studies (41 cohorts; 11,346 patients) were included. Elevated pretreatment NLR was associated with poorer OS (HR = 1.68, 95% CI 1.54–1.84; P < 0.0001) and poorer progression- or recurrence-related survival (HR = 1.61, 95% CI 1.43–1.81; P < 0.0001). The direction of association was generally consistent across exploratory subgroups defined by treatment category, age, geographic region, and NLR cut-off. Leave-one-out analyses did not identify a single study that changed the direction of either pooled association. Between-study heterogeneity and evidence of small-study effects warrant cautious interpretation.

**Conclusion:**

Elevated pretreatment NLR was associated with poorer survival outcomes in chemotherapy-treated CRC. NLR may be a readily available prognostic biomarker, but heterogeneous populations, variable outcome definitions and cut-off values, inconsistent covariate adjustment, and possible small-study effects currently limit its use for clinical decision-making. Prospective validation and methodological standardization are required before routine application.

**Systematic Review Registration:**

https://www.crd.york.ac.uk/PROSPERO/, identifier CRD420251136904.

## 1 Introduction

Globally, colorectal cancer (CRC) ranks as the third most frequently diagnosed cancer and the second leading cause of cancer death, contributing to nearly 10% of all cancer diagnoses. Notably, its incidence is increasing among younger populations (1). Recent statistics confirm that CRC remains a major burden for global healthcare systems, driven by consistently high rates of incidence and mortality (2).

Surgical resection remains the principal curative treatment for localized CRC, whereas chemotherapy is used across adjuvant, neoadjuvant, and palliative settings and is central to the management of advanced or metastatic disease (3). These settings differ in disease burden, treatment intent, concurrent radiotherapy or targeted therapy, and host inflammatory status. Such heterogeneity creates a need for inexpensive markers that can support prognostic assessment without being interpreted as treatment-selection biomarkers.

Systemic inflammation contributes to cancer development and progression and is closely related to antitumor immune function (4, 5). The neutrophil-to-lymphocyte ratio (NLR), calculated from a routine complete blood count, reflects the balance between circulating neutrophils and lymphocytes and has attracted interest because it is inexpensive and widely available. Multiple studies of chemotherapy-treated CRC have associated elevated pretreatment NLR with shorter overall survival (OS) and progression-free, disease-free, or recurrence-free survival outcomes (5). These observations support a prognostic association, but they do not establish that NLR predicts differential benefit from a specific treatment.

Previous meta-analyses evaluated NLR across broad CRC populations that included surgically treated patients, patients receiving systemic therapy, and mixed treatment settings. A chemotherapy-focused synthesis may provide additional clinical context because disease burden, treatment intent, treatment-related hematologic effects, radiotherapy, and concurrent targeted agents may modify the association between systemic inflammation and survival. Earlier reviews also predated several recent cohorts and provided limited assessment of treatment setting, age, geographic region, and NLR cut-off as potential contributors to heterogeneity (6, 7).

We therefore conducted a systematic review and meta-analysis focused on CRC patients receiving chemotherapy. The objectives were to estimate the association between pretreatment NLR and survival outcomes and to explore whether treatment category, age, geographic region, and NLR cut-off might contribute to between-study heterogeneity. Because the included evidence was predominantly retrospective and used variable thresholds and outcome definitions, the analysis was intended to assess prognostic association rather than establish a clinical decision threshold.

## 2 Materials and methods

### 2.1 Literature search

This study adhered to the PRISMA 2020 guidelines (8), and its protocol was registered in the PROSPERO database (CRD420251136904). Two independent investigators (CWK and ZYH) developed the search strategy, which included subject headings and keywords tailored to multiple electronic databases: PubMed, Embase, Web of Science, and the Cochrane Library. Records were retrieved covering the entire period from the inception of the databases to July 15, 2025. Key search terms covered concepts related to neutrophils (e.g., “Neutrophils”, “Polymorphonuclear Leukocyte”, “LE Cell”), lymphocytes (e.g., “Lymphocytes”, “Lymphoid Cells”), their ratio, colorectal neoplasms (e.g., “Colorectal Cancer”, “Colorectal Tumors”), and treatments (e.g., “Chemotherapy”, “Pharmacotherapy”, “Drug Therapy”). Synonyms and variant forms of each term were incorporated to ensure comprehensive retrieval. **Supplementary Table S1** outlines the approach used in the literature search.

### 2.2 Study selection

Eligibility criteria were as follows: (a) pathologically confirmed colorectal cancer; (b) treatment involving chemotherapy, alone or combined with surgery, radiotherapy, or targeted therapy; (c) evaluation of pretreatment NLR in relation to OS or a secondary progression- or recurrence-related time-to-event outcome, including PFS, DFS, RFS, or an analogous endpoint as defined by the original study; (d) availability of an HR and corresponding 95% CI, either reported directly or calculable from the reported estimate; (e) comparison of high- and low-NLR groups using a stated cut-off; and (f) full-text publication in a peer-reviewed journal. Exclusion criteria were non-original publications, conference abstracts, case reports, letters, reports without extractable survival data, and duplicate or overlapping publications.

Two investigators (CWK and ZYH) independently screened the titles and abstracts of retrieved records. Potentially relevant studies underwent full-text review to determine eligibility. Any discrepancies arising during the selection process were resolved through discussion until a consensus was reached.

### 2.3 Data extraction

Two investigators (CWK and ZYH) independently extracted the first author, publication year, country, study design, sample size, age, study period, treatment setting, timing of NLR measurement, NLR cut-off, follow-up, outcome definition, and HR with its 95% CI. Disagreements were resolved by discussion. Effect estimates were used as reported in the source publications. The retained dataset contained both adjusted and unadjusted estimates because covariate adjustment was not reported consistently across studies. We therefore could not characterize the pooled HRs as primarily adjusted or primarily unadjusted, and an adjusted-only sensitivity analysis could not be reliably reconstructed from the retained study-level dataset. HRs were analyzed on the logarithmic scale, and standard errors were derived from the reported 95% CI limits. The analysis used aggregate study-level survival estimates rather than individual participant data.

### 2.4 Quality assessment

We employed the Newcastle–Ottawa Scale (NOS) to evaluate the methodological quality of the studies included in this meta-analysis. Each study was assessed across three domains—selection, comparability, and outcome—with a maximum possible score of 9 (9). For descriptive purposes, scores of 7–9 were classified as higher quality and scores below 7 as lower quality; this threshold was not used to exclude studies. Because NOS ratings can be sensitive to operationalization, they were interpreted alongside study design and control of confounding rather than as a definitive measure of validity (10).

### 2.5 Statistical analysis

Pooled HRs and 95% CIs were calculated on the log-HR scale using DerSimonian–Laird random-effects models because clinical and methodological heterogeneity was anticipated across disease stages and treatment settings. Heterogeneity was evaluated with Cochran’s Q test and quantified using the I² statistic. Exploratory subgroup analyses were conducted by treatment category, mean or median age, geographic region, and NLR cut-off; subgroup-specific estimates were interpreted descriptively because formal interaction tests were not available. Leave-one-out analyses examined the influence of individual studies. Funnel plots and Egger’s regression test were used to assess small-study effects when sufficient studies were available. Egger’s test was not interpreted as proof of publication bias. A trim-and-fill analysis was not performed because funnel asymmetry may reflect clinical and outcome heterogeneity rather than missing studies, making the missing-study assumption uncertain in this dataset. Statistical significance was defined as P < 0.05. Analyses were conducted using STATA 15.0 and RevMan 5.4 (11).

## 3 Results

### 3.1 Study characteristics

The database searches identified 1,332 records: 291 from PubMed, 659 from Embase, 48 from the Cochrane Library, and 334 from Web of Science. After removal of 404 duplicates, 928 records underwent title and abstract screening, and 881 were excluded. Forty-seven reports were sought for retrieval; seven could not be retrieved. Of the 40 full-text reports assessed for eligibility, two were excluded because the relevant outcome could not be extracted. Thirty-eight studies comprising 11,346 patients were included in the final meta-analysis (12–49) (**Figure 1**).

**Figure 1:**
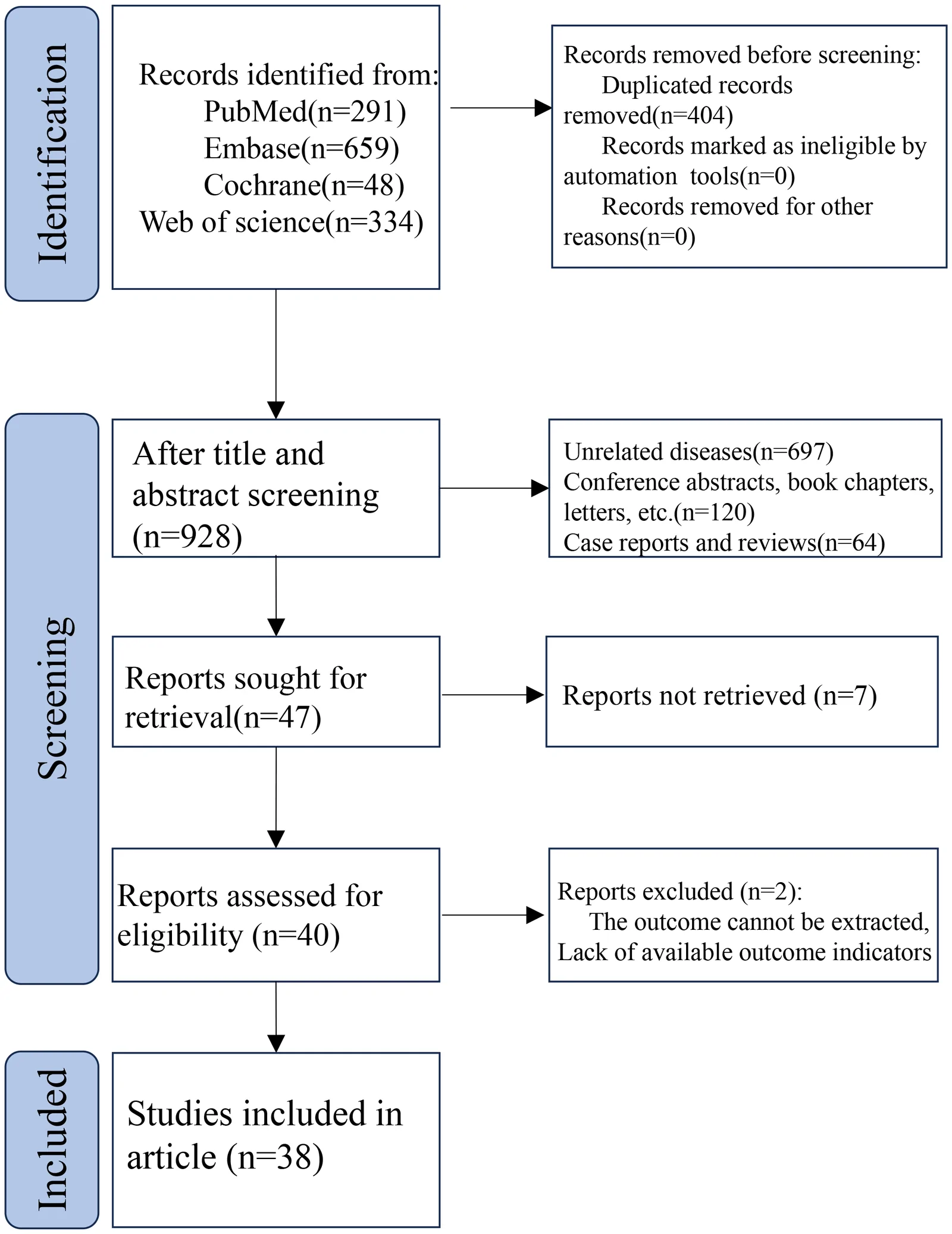
Flow chart of literature screening.

The 38 eligible studies contributed 41 reported cohorts. The evidence was predominantly retrospective, although it also included prospective cohorts and secondary analyses of trial datasets, and was published in English between 2009 and 2025. The populations covered different disease stages and treatment intents, including adjuvant or neoadjuvant chemoradiotherapy and systemic treatment for advanced or metastatic disease. Some cohorts received chemotherapy alone, whereas others included radiotherapy, surgery, or targeted agents such as bevacizumab or cetuximab. Thirty-seven cohort estimates contributed to the OS synthesis and 30 contributed to the secondary progression- or recurrence-related survival synthesis. **Table 1** summarizes the included studies.

**Table 1:** Basic characteristics of the included literature.

Author	Year	Source of patients	Study type	Duration	Age	Sample size	Treatment	NLR cut-off	Outcome	Quality score
Mao et al. (26)	2019	China	Retrospective	2006-2015	NA	183	chemotherapy with or without bevacizumab or cetuximab	2.3	OS	8
Petracci et al. (12)	2024	Italy	Retrospective	2007-2016	65	113	chemotherapy with bevacizumab	1.1	PFS/DFS/RFS	9
Petracci et al. (12)	2024	Italy	Retrospective	2007-2016	65	126	chemotherapy	1.1	PFS/DFS/RFS	9
Shen et al. (13)	2014	China	Retrospective	2006-2011	55	199	chemotherapy and chemoradiotherapy	2.8	OS,PFS/DFS/RFS	8
Kishi et al. (14)	2009	America	Retrospective	1997-2007	57	200	chemotherapy and resection	5	OS	7
Kishi et al. (14)	2009	America	Retrospective	1997-2007	56	90	chemotherapy	5	OS	7
Li et al. (15)	2021	China	Retrospective	2014-2017	63	118	chemotherapy	2.02	OS	9
Kim et al. (16)	2018	Korea	Retrospective	2008-2014	62	503	chemotherapy with or without bevacizumab or cetuximab	3	OS,PFS/DFS/RFS	9
Grenader et al. (17)	2016	Britain	Retrospective	2005-2014	64	1604	chemotherapy	2.2	OS	9
Wood et al. (18)	2017	Britain	Retrospective	2011-2016	64	1603	chemotherapy with or without bevacizumab	2.2	OS,PFS/DFS/RFS	9
García-Alfonso et al. (19)	2021	Spain	Retrospective	2019	65	402	chemotherapy including targeted agents	5	OS	7
Kaneko et al. (20)	2012	Japan	Retrospective	2005-2010	61	50	chemotherapy with oxaliplatin	4	OS,PFS/DFS/RFS	8
Ke et al. (21)	2020	China	Retrospective	2006-2016	63	184	chemotherapy	3.5	OS,PFS/DFS/RFS	8
Dias et al. (22)	2022	Portugal	Retrospective	2015-2018	62	178	chemotherapy	2.8	OS	8
Passardi et al. (24)	2016	Italy	Retrospective	2007-2012	65	148	chemotherapy	3	OS,PFS/DFS/RFS	8
Passardi et al. (24)	2016	Italy	Retrospective	2007-2012	65	141	chemotherapy with bevacizumab	3	OS,PFS/DFS/RFS	8
He et al. (25)	2013	China	Retrospective	2005-2010	56	243	chemotherapy	3	OS,PFS/DFS/RFS	8
Giakoustidis et al. (27)	2015	Britain	Retrospective	2005-2012	NA	169	chemotherapy	2.5	OS,PFS/DFS/RFS	9
Chua et al. (28)	2011	Australia	Retrospective	1999-2007	62	349	chemotherapy	5	OS,PFS/DFS/RFS	8
Jiang et al. (30)	2019	China	Retrospective	2010-2017	60	102	chemotherapy	3.285	OS,PFS/DFS/RFS	8
Yang et al. (31)	2018	China	Retrospective	2010-2015	53	98	chemotherapy	2.22	OS,PFS/DFS/RFS	8
Matsuda et al. (32)	2019	Japan	Retrospective	2014-2018	69	33	chemotherapy	5	OS,PFS/DFS/RFS	6
Sung et al. (33)	2017	Korea	Retrospective	2006-2013	59	110	chemotherapy and chemoradiotherapy	5.14	PFS/DFS/RFS	8
Dell’Aquila et al. (34)	2018	Italy	Retrospective	2014-2017	61	413	chemotherapy	3	OS,PFS/DFS/RFS	9
Nagasaki et al. (36)	2015	Japan	Retrospective	2004-2012	61	201	chemotherapy and chemoradiotherapy	3	OS,PFS/DFS/RFS	9
Yang et al. (38)	2019	China	Retrospective	2009-2015	56	220	chemotherapy	2.65	OS,PFS/DFS/RFS	7
Bulut et al. (39)	2022	Turkey	Retrospective	2010-2020	64	94	chemotherapy	3.44	OS,PFS/DFS/RFS	9
Cruz-Ramos et al. (40)	2019	Spain	Retrospective	2003-2015	72	110	chemotherapy	3.02	OS,PFS/DFS/RFS	7
Karaca et al. (41)	2024	Turkey	Retrospective	2016-2020	64	187	chemotherapy and chemoradiotherapy	3.63	OS,PFS/DFS/RFS	7
Miyamoto et al. (42)	2023	Japan	Retrospective	2005-2019	63	406	chemotherapy	3	OS	7
Nie et al. (44)	2025	China	Retrospective	2015-2018	54	116	chemotherapy	2.33	OS,PFS/DFS/RFS	6
Zhang et al. (45)	2020	China	Retrospective	2011-2015	56	472	chemotherapy	2.3	OS,PFS/DFS/RFS	8
Colloca et al. (46)	2021	Italy	Retrospective	2010-2015	69	128	first-line chemotherapy	2.3	OS,PFS/DFS/RFS	7
Colloca et al. (46)	2021	Italy	Retrospective	2010-2015	70	128	second-line chemotherapy	2.3	OS,PFS/DFS/RFS	7
Formica et al. (47)	2014	Italy	Retrospective	2008-2011	66	126	chemotherapy	5	OS	7
Carruthers et al. (48)	2012	Britain	Retrospective	2000-2005	63	115	chemotherapy and chemoradiotherapy	5	OS,PFS/DFS/RFS	7
Wang et al. (23)	2025	China	Retrospective	2013-2023	62	692	chemotherapy	2	OS	8
Jiang et al. (35)	2017	China	Retrospective	2007-2014	55	284	chemotherapy	2.47	OS,PFS/DFS/RFS	8
Clarke et al. (37)	2020	Australia	Prospective	2012-2016	64	128	chemotherapy with bevacizumab	5	OS,PFS/DFS/RFS	8
Azab et al. (49)	2014	America	Retrospective	2005-2011	68	580	chemotherapy and chemoradiotherapy	5	OS,PFS/DFS/RFS	8

OS, overall survival; PFS, progression-free survival; DFS, disease-free survival; RFS, recurrence-free survival; NLR, neutrophil-to-lymphocyte ratio; NA, not available. PFS/DFS/RFS denotes the study-specific progression- or recurrence-related endpoint used in the secondary synthesis.

Newcastle–Ottawa Scale scores ranged from 6 to 9. Most studies met the descriptive threshold for higher methodological quality, although the retrospective designs and variable control of confounding remained important limitations (**Supplementary Table S2**).

### 3.2 Meta-analysis results

#### 3.2.1 NLR and OS

The association between NLR and OS was evaluated across 37 cohort studies involving 10,997 participants. Given the considerable heterogeneity observed across studies, (*I^2^* = 54%, p < 0.0001; **Figure 2A**), a random-effects model was applied. Higher NLR values were significantly correlated with poorer overall survival among colorectal cancer patients treated with chemotherapy (HR = 1.68, 95% CI: 1.54–1.84; p < 0.0001; **Figure 2A**).

**Figure 2:**
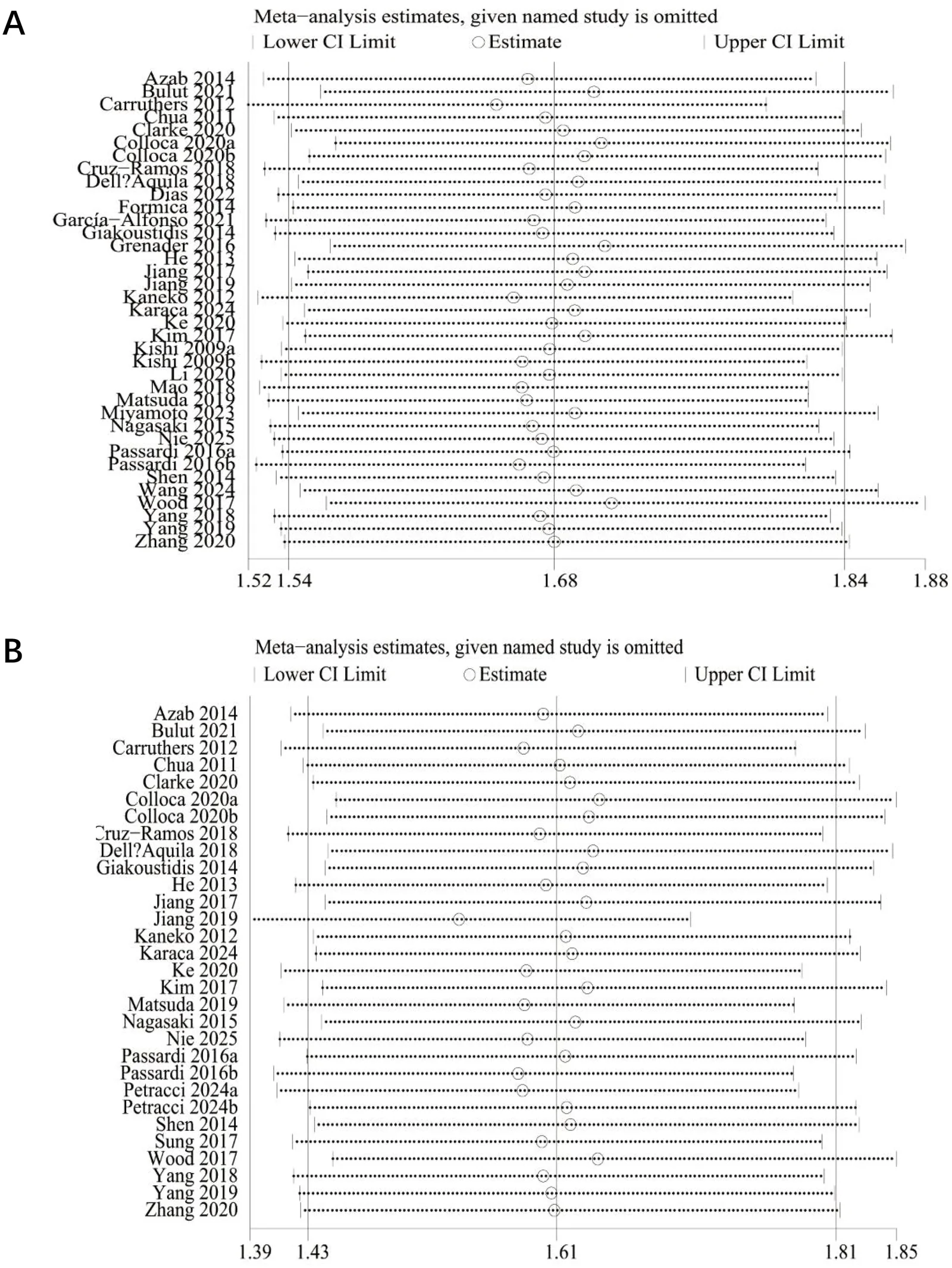
**(A)** Forest plot for the association between NLR and OS; **(B)** forest plot for the association between NLR and progression- or recurrence-related survival.

Exploratory subgroup analyses were performed according to treatment category, age, geographic region, and NLR cut-off (**Table 2**). The direction of association between elevated NLR and poorer OS was generally consistent across the reported subgroups. Treatment categories included chemotherapy alone, chemoradiotherapy, and chemotherapy with targeted therapy; several mixed or other treatment settings could not be represented fully by these categories. Estimates from subgroups containing few studies should be interpreted cautiously.

**Table 2:** Pooled HRs for OS and progression- or recurrence-related survival outcomes in subgroup analyses. The American subgroup for the secondary outcome included one study; between-study heterogeneity was therefore not estimable.

Subgroup	OS				PFS/DFS/RFS			
	Study	HR [95%CI]	P value	I2	Study	HR [95%CI]	P value	I2
*Total*	37	1.68 [1.54, 1.84]	<0.00001	54%	30	1.61 [1.43, 1.81]	<0.00001	69%
Treatment								
Chemotherapy	24	1.58 [1.44, 1.73]	<0.00001	41%	21	1.57 [1.36, 1.81]	<0.00001	67%
Chemoradiotherapy	5	2.42 [1.47, 3.98]	0.0005	60%	6	1.69 [1.24, 2.29]	0.0008	39%
Chemotherapy with targeted therapy	3	1.82 [1.39, 2.37]	<0.00001	54%	2	2.48 [1.80, 3.43]	<0.00001	0%
Mean age								
≥60 years	26	1.63 [1.47, 1.80]	<0.0001	60%	21	1.60 [1.39, 1.84]	<0.00001	76%
< 60 years	9	1.73 [1.49, 2.02]	<0.0001	0%	8	1.67 [1.37, 2.02]	<0.00001	13%
Region								
Asia	18	1.72 [1.55, 1.91]	<0.00001	14%	15	1.76 [1.47, 2.11]	<0.00001	59%
Europe	16	1.55 [1.37, 1.76]	<0.00001	64%	14	1.44 [1.27, 1.63]	<0.00001	55%
America	3	2.37 [1.69, 3.33]	<0.00001	0%	1	1.91 [1.26, 2.90]	0.002	NA
NLR cut-off								
≥3	22	1.78 [1.59, 1.98]	<0.00001	42%	18	1.73 [1.47, 2.04]	<0.00001	65%
<3	15	1.50 [1.33, 1.68]	<0.00001	43%	12	1.41 [1.24, 1.59]	<0.00001	43%

Heterogeneity in the overall OS analysis was I² = 54%. Lower within-subgroup I² values were observed in some strata, including chemotherapy-only studies, patients younger than 60 years, and studies conducted in Asia or America. These patterns suggest possible contributions from treatment setting, age, and region but do not establish them as sources of heterogeneity because the subgroup sizes differed and formal tests of interaction were not available.

#### 3.2.2 NLR and progression- or recurrence-related survival

Thirty cohort estimates involving 7,347 participants contributed progression-free, disease-free, recurrence-free, or analogous time-to-event outcomes as defined in the original studies. Elevated NLR was associated with poorer progression- or recurrence-related survival (HR = 1.61, 95% CI 1.43–1.81; P < 0.0001; **Figure 2B**), with substantial heterogeneity (I² = 69%; P < 0.0001).

The direction of association was generally consistent across exploratory subgroups defined by treatment category, age, geographic region, and NLR cut-off. Lower within-subgroup heterogeneity in selected strata may indicate that clinical setting and threshold selection contributed to variability; however, these observations were descriptive, and the small number of studies in some subgroups limited comparison.

### 3.3 Sensitivity analysis

Leave-one-out sensitivity analyses showed that sequential omission of individual studies did not change the direction of the pooled association or move the pooled 95% CI across the null for either OS (**Figure 3A**) or the secondary progression- or recurrence-related survival outcome (**Figure 3B**). These analyses support stability with respect to single-study influence but do not address outcome heterogeneity, residual confounding, or small-study effects.

**Figure 3:**
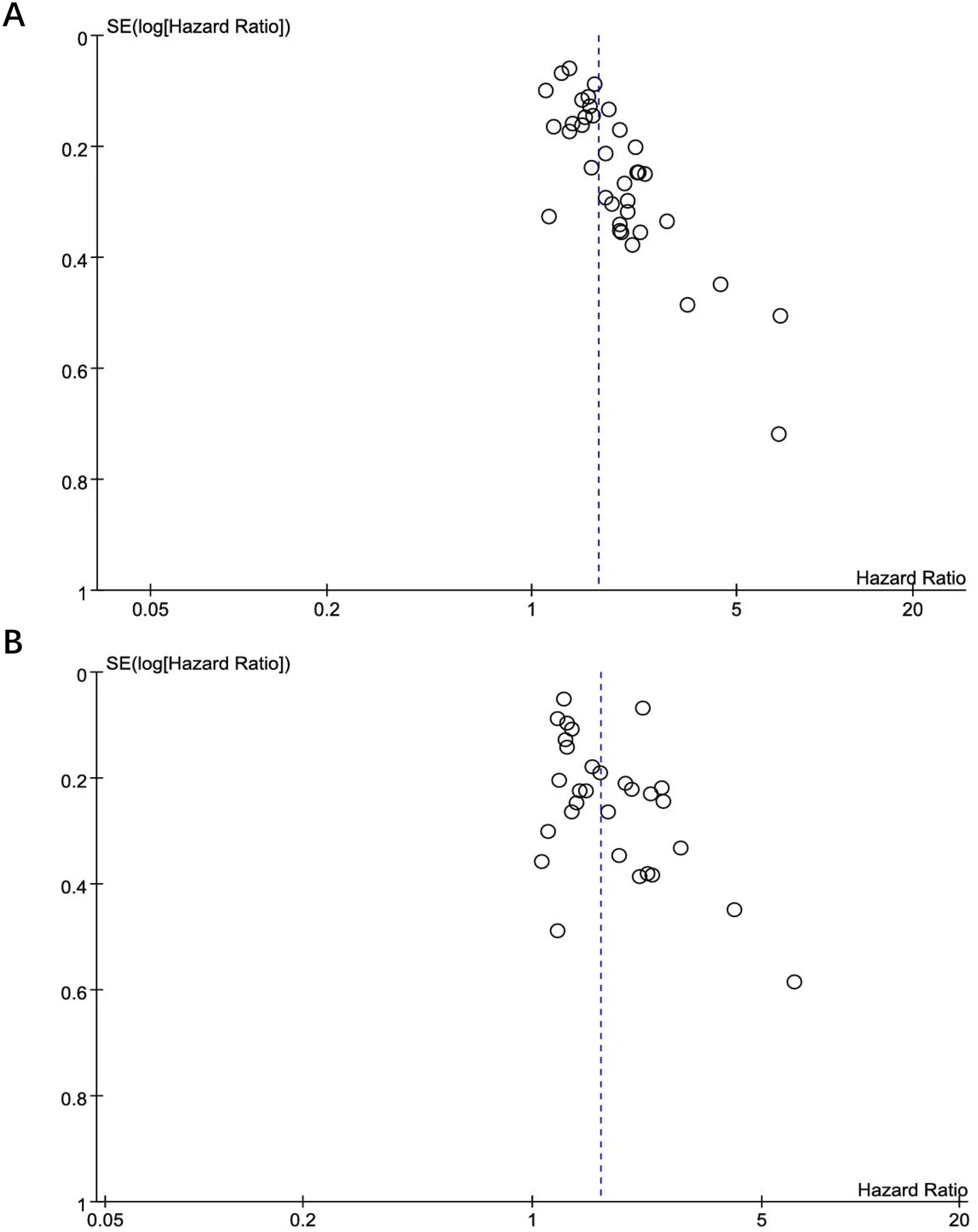
Leave-one-out sensitivity analysis of OS **(A)** and progression- or recurrence-related survival **(B)**.

### 3.4 Publication bias

Funnel plots (**Figure 4**) and Egger’s regression were used to assess small-study effects. Egger’s test indicated funnel-plot asymmetry for OS (P < 0.001) and for the secondary progression- or recurrence-related survival outcome (P = 0.045). The signal was stronger for OS. Although the funnel plots did not show a single simple pattern, visual inspection is subjective, and the statistical evidence of asymmetry warrants cautious interpretation. Funnel asymmetry may reflect publication bias, selective reporting, clinical heterogeneity, or other small-study effects.

**Figure 4:**
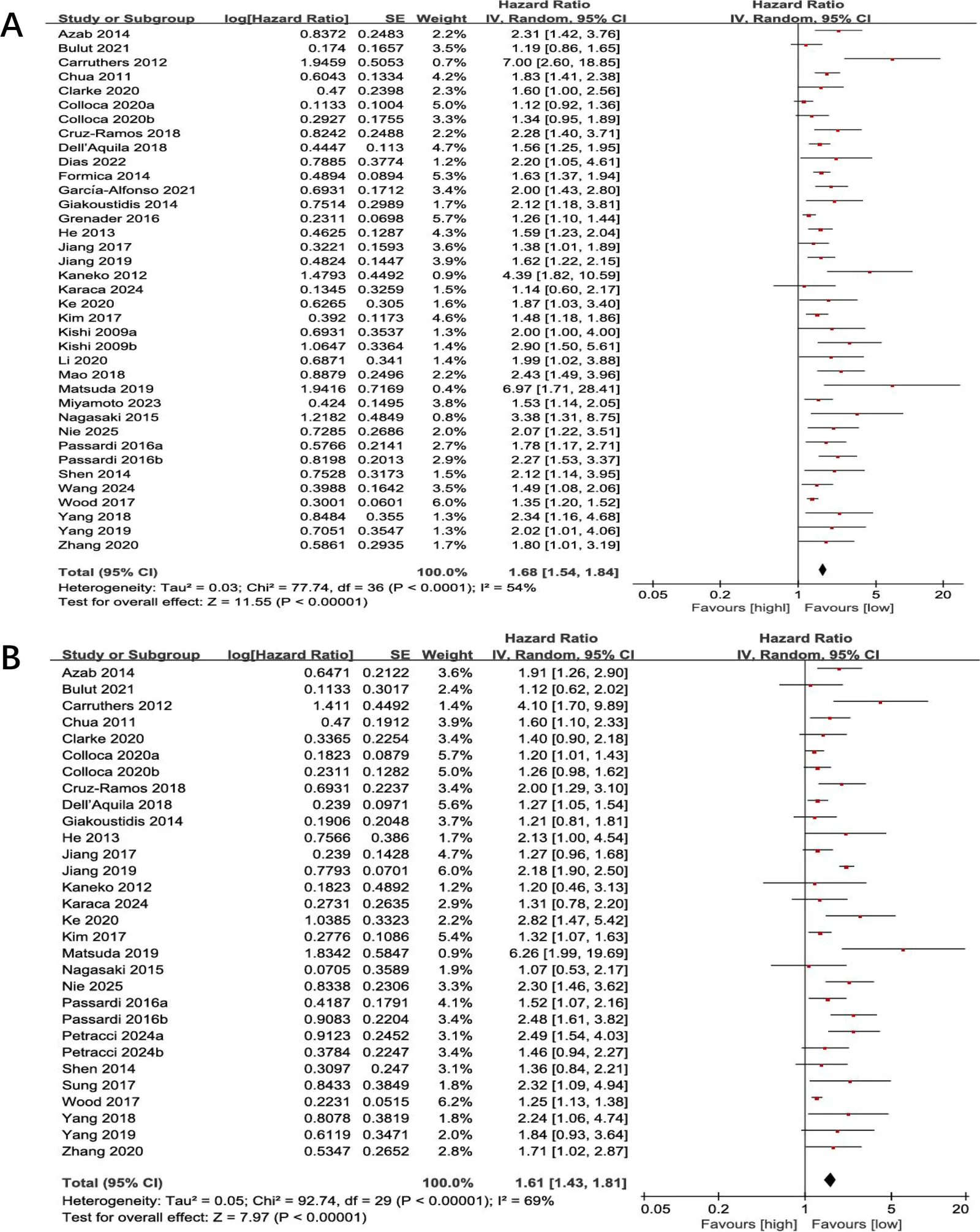
Funnel plots for OS **(A)** and progression- or recurrence-related survival **(B)**.

## 4 Discussion

This meta-analysis of 38 studies and 41 reported cohorts found that elevated pretreatment NLR was associated with poorer OS and poorer progression- or recurrence-related survival among chemotherapy-treated CRC patients. The pooled associations remained on the same side of the null in leave-one-out analyses. These findings support NLR as a potential prognostic biomarker, but they should not be interpreted as evidence of independence from other prognostic factors or as validation of a treatment-selection tool.

The principal incremental value of this review is its focus on patients receiving chemotherapy across adjuvant, neoadjuvant, and palliative settings. Earlier meta-analyses combined broader CRC populations undergoing surgery, local treatment, systemic therapy, or mixed management. A 2014 analysis of 13 cohorts and 4,056 patients reported an association between elevated NLR and poorer DFS/PFS across heterogeneous treatments (6), whereas a 2019 analysis of 16 surgically treated cohorts reported associations with OS, DFS, RFS, and disease-specific survival (7). By concentrating on chemotherapy-exposed populations and including studies published through 2025, the present analysis offers a treatment-contextualized estimate. This focus does not eliminate heterogeneity, because disease stage, treatment intent, metastatic status, tumor location, and concurrent radiotherapy or targeted therapy still differed substantially across studies.

Treatment setting may modify the observed prognostic association. Patients receiving neoadjuvant chemoradiotherapy for localized rectal cancer differ clinically from those receiving palliative systemic therapy for metastatic colon or rectal cancer. NLR has also been associated with outcomes in immunotherapy-treated advanced cancers (50), but that evidence arises from a distinct treatment context and cannot be assumed to apply directly to chemotherapy-treated CRC. Tumor burden, resection status, treatment line, molecular characteristics, primary tumor location, and exposure to bevacizumab or cetuximab may influence both baseline inflammation and survival. The exploratory treatment subgroups showed associations in the same direction, but several strata contained few studies, mixed treatment exposures were not fully separable, and no formal interaction test was available. The subgroup findings should therefore be viewed as hypothesis-generating rather than proof that the association is equivalent across treatment settings.

NLR thresholds varied widely among the included studies. Consequently, the pooled estimates compare study-specific “high” and “low” groups rather than validate a single universal clinical cut-off. The similar direction of association in studies using thresholds below 3 and those using thresholds of 3 or higher suggests that the prognostic signal was not confined to one threshold category, but it does not identify an optimal value. Differences in assay timing, acute infection, medication exposure, nutritional status, comorbidity, and treatment-related hematologic changes may also alter NLR. Standardized sampling conditions and prospectively defined, context-specific thresholds are needed before NLR can support routine clinical decisions.

NLR should also be considered within the broader field of blood-based inflammatory and nutritional biomarkers. A 2026 meta-analysis of the C-reactive-protein–albumin–lymphocyte (CALLY) index in CRC associated a higher CALLY index with better OS and DFS/RFS, while emphasizing the need for prospective validation and standardized cut-offs (51). Another 2026 meta-analysis in locally advanced rectal cancer found that higher pretreatment NLR, PLR, and systemic immune-inflammation index values were associated with lower odds of pathological complete response after neoadjuvant chemoradiotherapy (52). These composite indices may capture dimensions of inflammation and nutritional status that NLR alone does not. NLR nevertheless remains attractive because it requires only a routine complete blood count, is inexpensive, and is widely available; its simplicity must be balanced against biological variability and limited specificity.

Biologically, elevated NLR may reflect both neutrophil-predominant inflammation and reduced lymphocyte-mediated antitumor immunity. Tumor-associated neutrophils can promote angiogenesis, invasion, and immunosuppression, whereas reduced or dysfunctional lymphocyte responses may weaken tumor control (53–55). Inflammatory signaling pathways may further connect systemic inflammation with tumor progression and treatment resistance (56, 57). These mechanisms are plausible but do not establish that NLR itself is causal or that modifying NLR would improve outcomes.

The findings therefore support NLR as a low-cost candidate prognostic biomarker rather than an established clinical decision marker. The analysis did not test treatment-by-NLR interactions and cannot determine whether patients with different NLR values derive differential benefit from specific regimens. NLR may complement, but should not replace, established factors such as stage, performance status, molecular characteristics, nutritional status, tumor location, and treatment response.

Several limitations warrant emphasis. First, most evidence was retrospective or derived from secondary analyses of existing cohorts or trials, with risks of selection bias and residual confounding. Second, the pooled estimates comprised a mixture of adjusted and unadjusted HRs, and covariates were not standardized across studies; independence from other prognostic factors therefore cannot be inferred. Third, disease stage, metastatic status, tumor location, treatment intent, treatment line, and concurrent radiotherapy or targeted therapy varied substantially. Fourth, the secondary synthesis combined PFS, DFS, RFS, and analogous outcomes as defined by the original studies; these endpoints are clinically related but not interchangeable. Fifth, NLR measurement conditions and cut-offs were inconsistent. Sixth, Egger’s tests suggested small-study effects for both outcomes, particularly OS, and publication or selective-reporting bias cannot be excluded. A trim-and-fill analysis was not performed because its missing-study assumptions were difficult to separate from the substantial clinical and outcome heterogeneity; the absence of this sensitivity analysis is itself a limitation. Finally, restriction to English-language published reports may have excluded relevant evidence.

In conclusion, elevated pretreatment NLR was associated with poorer survival outcomes in chemotherapy-treated CRC. Its accessibility and low cost make it a promising prognostic biomarker, but the current evidence does not support a universal threshold or immediate use for treatment selection. Prospective multicenter studies with standardized sampling, outcome definitions, cut-offs, and covariate adjustment are required before routine clinical application.

## Data Availability

This meta-analysis used data extracted from previously published studies. The original sources are cited in the article.
