# Supplementary material for: Prognostic value of the neutrophil-to-lymphocyte ratio in patients with colorectal cancer undergoing chemotherapy: a systematic review and meta-analysis
Source: Front Oncol. 2026 Sep 28;16:1945549. doi: 10.3389/fonc.2026.1945549 (PMC13654844; doi:10.3389/fonc.2026.1945549)
Supplement: Supplementary file 1 [file Table1.docx]

TableS1 Literature search strategy

Pubmed-291

((((("Neutrophils"[Mesh]) OR (((Neutrophil) OR (Polymorphonuclear Leukocyte)) OR (LE Cell))) AND (("Lymphocytes"[Mesh]) OR (((Lymphocyte) OR (Lymphoid Cells)) OR (Lymphoid Cell)))) AND (Ratio)) AND (("Colorectal Neoplasms"[Mesh]) OR (((Colorectal Neoplasm) OR (Colorectal Tumors)) OR (Colorectal Cancer)))) AND (("Drug Therapy"[Mesh]) OR (((Chemotherapy) OR (Pharmacotherapy)) OR (Drug Therapies)))

Embase-659

((Neutrophils or (Neutrophil or Polymorphonuclear Leukocyte or LE Cell)) and (Lymphocytes or (Lymphocyte or Lymphoid Cells or Lymphoid Cell)) and Ratio and (Colorectal Neoplasms or (Colorectal Neoplasm or Colorectal Tumors or Colorectal Cancer)) and (Drug Therapy or (Chemotherapy or Pharmacotherapy or Drug Therapies))).af.

Cochrane-48

((Neutrophils or (Neutrophil or Polymorphonuclear Leukocyte or LE Cell)) and (Lymphocytes or (Lymphocyte or Lymphoid Cells or Lymphoid Cell)) and Ratio and (Colorectal Neoplasms or (Colorectal Neoplasm or Colorectal Tumors or Colorectal Cancer)) and (Drug Therapy or (Chemotherapy or Pharmacotherapy or Drug Therapies))).af.

WOS-334

(((((Neutrophils) OR (((Neutrophil) OR (Polymorphonuclear Leukocyte)) OR (LE Cell))) AND ((Lymphocytes) OR (((Lymphocyte) OR (Lymphoid Cells)) OR (Lymphoid Cell)))) AND (Ratio)) AND ((Colorectal Neoplasms) OR (((Colorectal Neoplasm) OR (Colorectal Tumors)) OR (Colorectal Cancer)))) AND ((Drug Therapy) OR (((Chemotherapy) OR (Pharmacotherapy)) OR (Drug Therapies))) (Topic)

Table S2. Quality evaluation of the eligible studies with Newcastle–Ottawa scale.

| Study | Selection | | | | Comparability | | Outcome | | |
| --- | --- | --- | --- | --- | --- | --- | --- | --- | --- |
|  | Representative-ness | Selection of  non-exposed | Ascertainment  of exposure | Outcome not present at start | Comparability on most important factors | Comparability on other risk factors | Assessment of outcome | Long enough follow-up (median≥1 year) | Adequacy  (completeness) of follow-up |
| Mao 2018 | * | * | * | * | * | - | * | * | * |
| Petracci 2024 | * | * | * | * | * | * | * | * | * |
| Shen 2014 | * | * | * | * | * | - | * | * | * |
| Kishi 2009 | * | * | * | * | - | - | * | * | * |
| Li 2020 | * | * | * | * | * | * | * | * | * |
| Kim 2017 | * | * | * | * | * | * | * | * | * |
| Grenader 2016 | * | * | * | * | * | * | * | * | * |
| Wood 2017 | * | * | * | * | * | * | * | * | * |
| García-Alfonso 2021 | * | * | * | * | * | - | * | - | * |
| Kaneko 2012 | * | * | * | * | * | - | * | * | * |
| Ke 2020 | * | * | * | * | * | - | * | * | * |
| Dias 2022 | * | * | * | * | * | - | * | * | * |
| Passardi 2016 | * | * | * | * | * | - | * | * | * |
| He 2013 | * | * | * | * | * | - | * | * | * |
| Giakoustidis 2015 | * | * | * | * | * | * | * | * | * |
| Chua 2011 | * | * | * | * | * | - | * | * | * |
| Jiang 2019 | * | * | * | * | * | - | * | * | * |
| Yang 2018 | * | * | * | * | * | - | * | * | * |
| Matsuda 2019 | - | * | * | * | * | - | * | - | * |
| Sung 2017 | * | * | * | * | * | - | * | * | * |
| Dell’Aquila 2018 | * | * | * | * | * | * | * | * | * |
| Nagasaki 2015 | * | * | * | * | * | * | * | * | * |
| Yang 2019 | - | * | * | * | * | - | * | * | * |
| Bulut 2021 | * | * | * | * | * | * | * | * | * |
| Cruz-Ramos 2018 | - | * | * | * | * | - | * | * | * |
| Karaca 2024 | - | * | * | * | * | - | * | * | * |
| Miyamoto 2023 | - | * | * | * | * | - | * | * | * |
| Nogueira-Costa 2020 | * | * | * | * | * | - | * | * | * |
| Nie 2025 | - | * | * | * | - | - | * | * | * |
| Zhang 2020 | * | * | * | * | * | - | * | * | * |
| Colloca 2021 | - | * | * | * | * | - | * | * | * |
| Formica 2014 | - | * | * | * | * | - | * | * | * |
| Carruthers 2012 | * | * | * | * | - | - | * | * | * |
| Wang 2024 | * | * | * | * | * | - | * | * | * |
| Chen 2022 | - | * | * | * | * | - | * | * | * |
| Jiang 2017 | * | * | * | * | * | - | * | * | * |
| Clarke 2020 | * | * | * | * | * | - | * | * | * |
| Azab 2014 | * | * | * | * | * | - | * | * | * |

*indicates criterion met; - indicates significant of criterion not met.
